# Propulsion Phase of the Single Leg Triple Hop Test in Women with Patellofemoral Pain Syndrome: A Biomechanical Study

**DOI:** 10.1371/journal.pone.0097606

**Published:** 2014-05-15

**Authors:** Andre Serra Bley, João Carlos Ferrari Correa, Amir Curcio Dos Reis, Nayra Deise Dos Anjos Rabelo, Paulo Henrique Marchetti, Paulo Roberto Garcia Lucareli

**Affiliations:** 1 Department of Rehabilitation Science, Human Motion Analysis Laboratory, Universidade Nove de Julho, São Paulo, Brazil; 2 Post Graduation Program in Human Movement Science, Universidade Metodista de Piracicaba, UNIMEP, Piracicaba, São Paulo, Brazil; 3 Faculty of Physical Education (YMCA), Sorocaba, São Paulo, Brazil; University of Sao Paulo, Brazil

## Abstract

Asymmetry in the alignment of the lower limbs during weight-bearing activities is associated with patellofemoral pain syndrome (PFPS), caused by an increase in patellofemoral (PF) joint stress. High neuromuscular demands are placed on the lower limb during the propulsion phase of the single leg triple hop test (SLTHT), which may influence biomechanical behavior. The aim of the present cross-sectional study was to compare kinematic, kinetic and muscle activity in the trunk and lower limb during propulsion in the SLTHT using women with PFPS and pain free controls. The following measurements were made using 20 women with PFPS and 20 controls during propulsion in the SLTHT: kinematics of the trunk, pelvis, hip, and knee; kinetics of the hip, knee and ankle; and muscle activation of the gluteus maximus (GM), gluteus medius (GMed), biceps femoris (BF) and vastus lateralis (VL). Differences between groups were calculated using three separate sets of multivariate analysis of variance for kinematics, kinetics, and electromyographic data. Women with PFPS exhibited ipsilateral trunk lean; greater trunk flexion; greater contralateral pelvic drop; greater hip adduction and internal rotation; greater ankle pronation; greater internal hip abductor and ankle supinator moments; lower internal hip, knee and ankle extensor moments; and greater GM, GMed, BL, and VL muscle activity. The results of the present study are related to abnormal movement patterns in women with PFPS. We speculated that these findings constitute strategies to control a deficient dynamic alignment of the trunk and lower limb and to avoid PF pain. However, the greater BF and VL activity and the extensor pattern found for the hip, knee, and ankle of women with PFPS may contribute to increased PF stress.

## Introduction

Patellofemoral pain syndrome (PFPS) is one of the most common musculoskeletal problems seen in orthopedic practice and accounts for approximately 25% of knee conditions [1]. PFPS often affects young, active women [2], 70% of cases involve women who are between 16 and 25 years of age [3] and is characterized by anterior knee pain that increases after prolonged sitting and during activities involving a high degree of quadriceps activity [4], [5]. The pain is the result of an imbalance in force distribution in the patellofemoral (PF) joint [6], which leads to an increase in local intraosseous pressure [7].

The etiology and progression of PFPS are commonly associated with elevated PF stress [8], which may be the result of “poor dynamic alignment” of the lower limb in weight-bearing activities. Excessive contralateral pelvic drop, hip internal rotation and adduction, knee valgus and ankle pronation lead to smaller areas of contact and consequently increase the pressure on the PF joint [9]–[11]. Studies using cadavers simulating weight-bearing activities on the lower limb have demonstrated an elevated PF stress in the presence of hip and knee kinematic abnormalities, which may contribute to the symptoms found in patients with PFPS [12], [13].

Recent kinematic studies have associated PFPS with asymmetry in lower limbs alignment during weight-bearing activities such ascending and descending stairs, squatting and landing [5], [14]–[17]. Abnormal trunk and lower limb frontal plane biomechanics [18], associated with impaired gluteus medius activation [18], [19], and an increased load on the hamstrings [20] may contribute to elevated PF stress in women with PFPS.

Activities with greater mechanical demands could be influenced by the external joint moments generated, which may lead to inadequate mechanics of the lower limb [14], [16]. The single-leg triple-hop test (SLTHT) is a challenging functional assessment that includes propulsion and landing phases. It is widely used in clinical practice and a reliable and reproducible method of clinically detecting differences between the rehabilitation period and the discharge criteria of patients with knee injuries [21]. It is also used to assess knee dynamic stabilization [22]. High neuromuscular demands are placed on the lower limb to achieve the maximum distance performance while performing this test [23]–[26]. Nevertheless, performance related to to biomechanics variables of healthy individuals and those with PFPS remain unclear. Therefore, this test requires further study.

Most biomechanical studies of the lower limbs [11], [15], [27]–[30] elected to assess the landing phase of the jump because they supposed that it involves greater mechanical power absorption and that abnormal alignment of the lower limbs may increase patellofemoral stress. The propulsion phase of the SLTHT generates sufficient knee joint power to achieve the aim of this test and, together with possible alterations in the kinematic alignment of the lower limbs, may affect the pathomechanics of PFPS. To our knowledge, biomechanical characteristics during hop tests have not yet been investigated in individuals with PFPS and no studies were found on the biomechanical behavior of the trunk and lower limb during the propulsion phase of hop or functional tests. Given the limited amount of biomechanical studies focusing on functional performance tests, the aim of the present study was to compare (1) the trunk, pelvis, hip, knee and ankle kinematics, (2) the hip, knee and ankle internal moments generated on the frontal and sagittal planes during peak knee flexion and (3) hip and knee neuromuscular control during the propulsion of the SLTHT in women with and without PFPS.

We hypothesized that, in comparison to the control group, women with PFPS would exhibit the following: greater ipsilateral trunk lean; contralateral pelvic drop; hip adduction and internal rotation; ankle pronation; greater internal hip abductor and extensor, knee adductor, ankle plantarflexor and supinator internal joint moments. We also hypothesized that women with PFPS would exhibit greater activation of the gluteus medius (GMed) and gluteus maximus (GM) muscles, in conjunction with lower activation of the vastus lateralis (VL) and biceps femoris (BF), during the propulsion phase of the SLTHT.

## Methods

### Subjects

A cross-sectional study was carried out involving 40 women, due to kinematic gender differences [29], who were aged between 18 and 35 years and physically active (at least 20 minutes of physical activity 3 times a week) [30]. The women were allocated into two groups: those with PFPS in the patellofemoral group (PFG), n = 20; and healthy controls in the control group (CG), n = 20. The groups were matched for age, weight, height and body mass index, with differences regarding anterior knee pain, as scored using a visual analogue scale (VAS) [25] and the anterior knee pain scale (AKPS) [31], [32] (Table 1).

**Table 1:** Characteristics of groups.

	Control group (n = 20)	Patellofemoral group (n = 20)	*P-*value
Age (years)	23.1 (3.3)	23.5 (2.1)	0.719
Body mass (kg)	55.9 (7.1)	55.3 (4.8)	0.821
Height (m)	1.62 (0.06)	1.65 (0.04)	0.205
Body mass index (Kg/m^2^)	21.3 (2.7)	20.2 (1.8)	0.233
Pain score (VAS)	0	4.9 (1.6)	0.001
Functional capacity (AKPS)	99.5 (1.2)	80.2 (4.9)	0.001

Data expressed as mean (standard deviation). Abbreviations: VAS =  Visual analog scale; AKPS =  anterior knee pain scale.

The PFG met the following inclusion criteria: a history of anterior knee pain for more than three months associated with a pain increase while performing at least two of the following activities: ascending or descending stairs; squatting; jumping; running and prolonged sitting [33]; pain intensity in the previous week scoring at least 3 points on the 0–10 VAS, on which 0 indicates no pain and 10 indicates the worst imaginable pain. The exclusion criteria were the following: neurological disorders, cardiovascular problems, a history of surgery or musculoskeletal injuries in the lower limbs or trunk, participating in rehabilitation programs for any musculoskeletal disorders, lower limb discrepancy greater than 1 cm, use of analgesics and current pregnancy.

All subjects were recruited from a local physiotherapy clinic and common areas of the university by a licensed physiotherapist with more than 10 years of experience. Eligible subjects were informed of the details of the study and gave written consent prior to participation. The present study was approved by the Human Research Ethics Committee of the *Universidade Nove de Julho* under protocol number 15426/2012.

### Instrumentation

Three-dimensional kinematic analyses of the trunk and lower limb were performed using a system with eight infrared cameras (SMART-D BTS, Milan, Italy), sampling at 100 Hz, to detect 25 reflective spherical markers attached to the skin with double-faced adhesive tape at the following locations: over the manubrium; xiphoid process; right scapula; acromions, 7^th^ cervical vertebra; 10^th^ thoracic vertebra; anterosuperior and posterosuperior iliac spines; side of thigh; lateral face of the base of the patella; lateral femoral epicondyle; side of shin; lateral malleolus; middle third of the foot between the 2^nd^ and 3^rd^ metatarsals and the calcaneus, based on the Vicon Plug-in Gait biomechanical model [34], [35]. The kinetic data were collected with a force plate (model 9286A, Kistler group, Winterthur, Switzerland) and sampling at 400 Hz.

A wireless hardware system (FREE EMG, BTS Bioengineering, Milan, Italy) was used for dynamic surface electromyography (EMG), with four analog inputs sampling at 1000 Hz per channel, detected using disposable, self-adhesive, differential, bipolar, Ag/AgCl surface electrodes measuring 1 cm in diameter (Medi-Trace 200 Kendall Healthcare/Tyco, Canada), spaced 2 cm from center to center, connected to a portable amplifier with a common-mode rejection ratio greater than 100 dB, output impedance exceeding 10 MΩ, cutoff frequency of 20 to 500 Hz and a gain of 1000x. Before electrode placement, the skin was shaved and abraded with alcohol. The surface electrodes were positioned at the midpoint between (1) the sacrum and the greater trochanter (GM), (2) the iliac crest and the greater trochanter (GMed), (3) the head of the fibula and ischial tuberosity (BF) and (4) in the lower third between the base of the patella and the anterosuperior iliac spine (VL) [36]. Surgical tape was placed over the electrodes to minimize movements that could cause artifacts in the signal readings. The EMG signal was digitalized by a 16-bit A/D converter and synchronized with the kinematic and kinetic data.

### Procedures

Subjects reported to the Motion Analysis Laboratory for a single testing session. The anthropometric data, VAS scores and AKPS scores were recorded first [32]. The affected leg (or most affected leg) was tested in the PFG. The correspondent limb of the CG participants was analyzed. The participants wore shorts and a top and were barefoot. Prior to the data collection, warm up exercises was performed on a treadmill for 10 minutes at 1.5 m/s.

**Figure 1:**
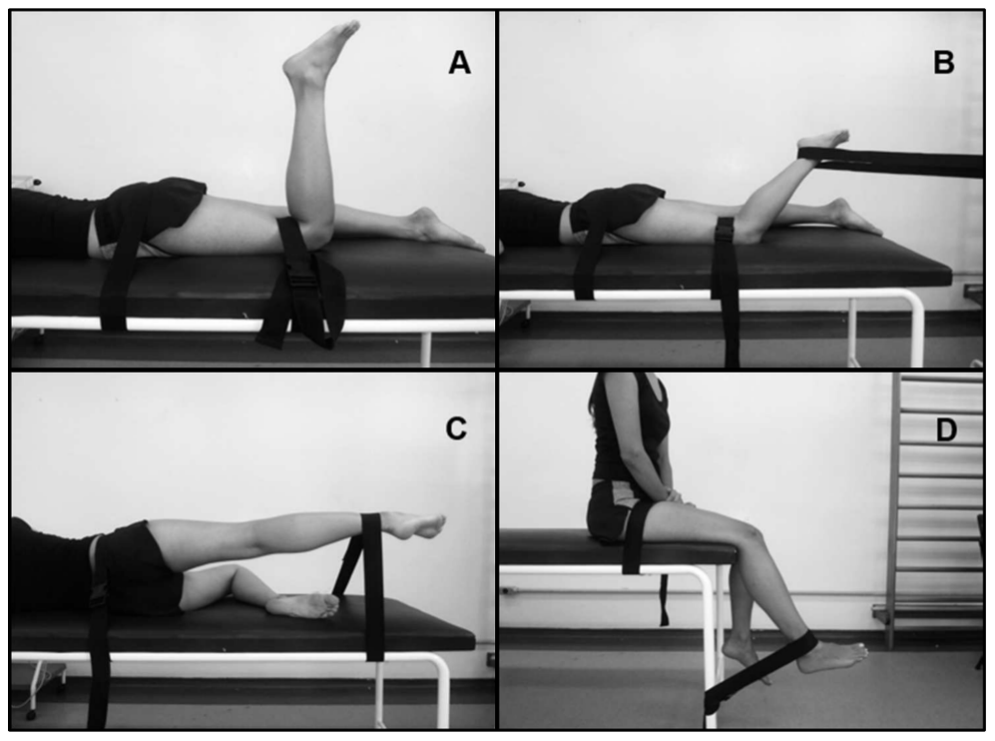
Surface electromyography during a maximum voluntary isometric contraction (MVIC). Positioning of the subjects performing a MVIC for the gluteus maximus (A), biceps femoris (B), gluteus medius (C) and vastus lateralis (D), against a fixed resistance.

In order to understand the contribution (EMG activation) made by the muscles, the EMG sign collected during the SLTHT was normalized for all subjects performing a maximum voluntary isometric contraction (MVIC) for the GM, BF, GMed, and VL, against a fixed resistance. Then, four trials of five-second MVICs were performed for each muscle, with a one-minute rest between contractions. The first MVIC was performed to familiarize the participant with the procedure. The patient was in the prone position, with the lower limb to be tested at 90° of knee flexion, resistance on the distal region of the thigh (GM MVIC) and the pelvis in a stabilized position. The hip was positioned in slight lateral rotation and the knee was flexed at 60°, with resistance on the distal region of the shin (BF MVIC). With the subject lying on her side, the knee was extended and the hip was placed in slight extension and abduction, with resistance on the shin distal region (GMed MVIC). Finally, they were placed in a sitting position with the knee flexed at 60° and resistance on the shin distal region (VL MVIC) (Figure 1). Verbal encouragement was given during all MVICs. The order of MVIC was counterbalanced to avoid any potential bias.

**Figure 2:**
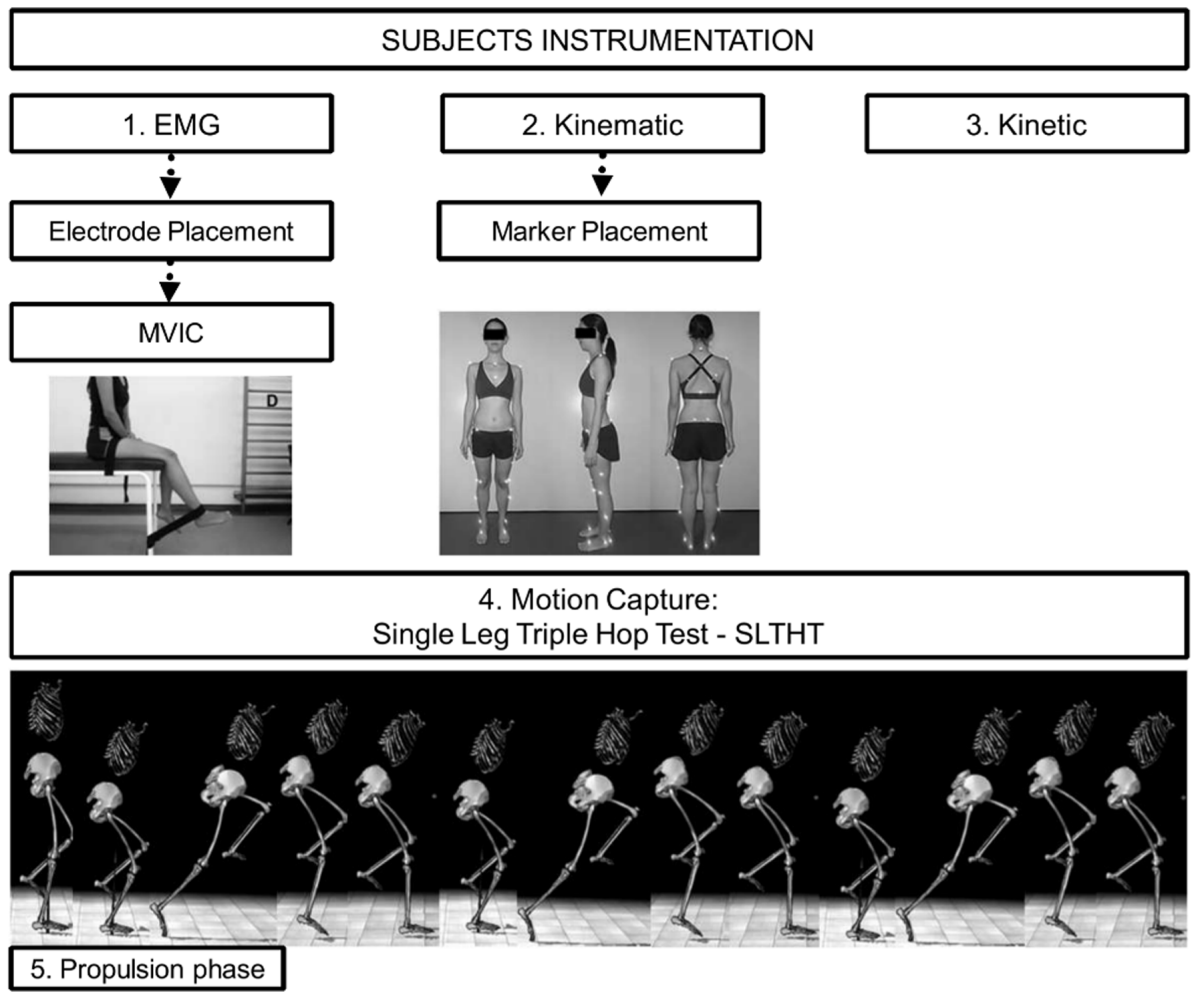
Subject instrumentation. 1. Electromyography (EMG) – electrode placement and subjects performed a maximum voluntary isometric contraction (MVIC); 2. Kinematic – marker placement; 3. Kinetic; 4. Motion capture during SLTHT – EMG, Kinematic and Kinetic data were captured simultaneously; 5. The propulsion phase was analysed.

Next, the subjects were familiarized with the SLTHT, which consists of three consecutive one-leg hops, the aim of which is to reach the maximum possible distance [37]. The analyzed movement comprised the SLTHT without upper-limb movement, which was excluded to avoid arm movements influencing the jump data [38], [39]. The arms were positioned with the elbows along the body and the hands placed on the chest. Once the patient felt comfortable with the execution, they were allowed to rest for two minutes and they then performed the SLTHT from a one-leg static standing position with the foot positioned over the force plate (3 times, with a one-minute rest period between tests) (Figure 2).

### Data analysis

**Figure 3:**
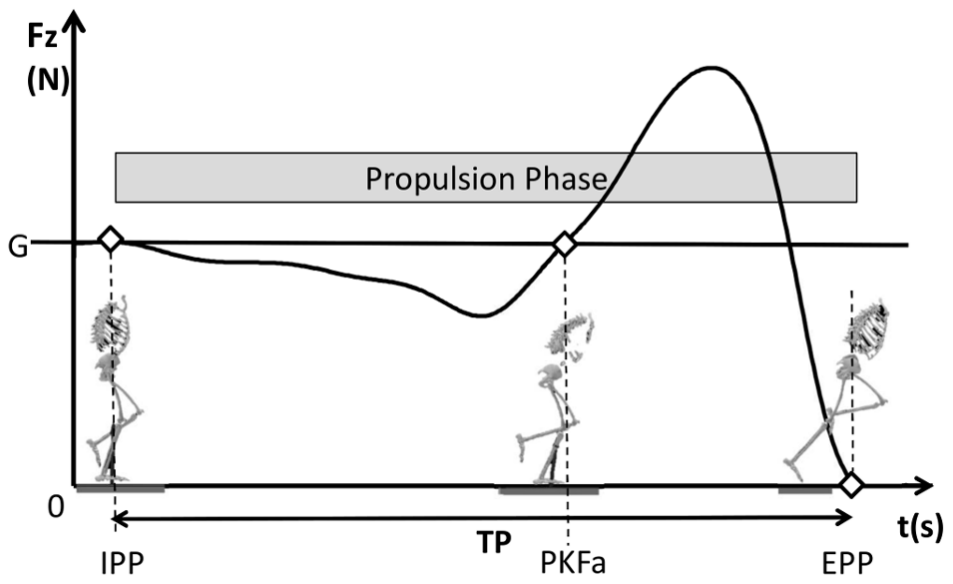
Time course of the ground reaction force during the propulsion phase of the single leg hop test (SLHT). Fz(t) – normalized vertical ground reaction force; IPP – initial propulsion phase; EPP – end propulsion phase; TP – duration of the propulsion phase; PKFa – Peak Knee Flexion Angle; G – gravitational force acting on the human body (G = m.g, where m is the mass of the subject and g is the gravitational acceleration).

The propulsion phase was defined as the instant at which the normalized vertical ground reaction force (vGRF) decreased from the body weight force and ended when the vGRF reached zero and foot took off (Figure 3). The peak joint angles for trunk flexion and lean, contralateral pelvic drop, hip flexion, adduction and internal rotation, knee flexion, ankle supination and dorsiflexion were obtained from the kinematic data. Internal joint moments for the hip, knee and ankle joints on the frontal and sagittal planes were determined through inverse dynamics (normalized by body mass) [32] and recorded at the peak knee flexion angle. Kinematic and kinetic data were converted to the C3D format using Matlab software (MathWorks, Inc, Natick, MA), applying the BTK 0.1.10 code (Biomechanical ToolKit) [40]. Marker trajectory and ground reaction force data were filtered using a Woltring filtering routine, with a 12 Hz cutoff frequency (Vicon Nexus software).

Muscle activity analysis was performed by a custom program in Matlab software (MathWorks, Inc, Natick, MA). Raw EMG signals were band-pass filtered at 20 to 400 Hz by a fourth-order Butterworth filter and smoothed using a 150-ms root mean square (RMS) sliding-window function. EMG data were normalized by the peak of the RMS value from MVIC and the EMG RMS data were then integrated (IEMG). The IEMG descending phase was defined as the interval between the vGRF decreasing from body weight force to peak knee flexion angle. IEMG data were expressed as the percentage of MVIC.s.

### Statistical analysis

The sample size calculation was based on peak knee flexion [41], in which the maximum amplitudes reached on the sagittal plane can influence the kinematics and kinetics of the lower limb. Considering a difference of 11° between group, a standard deviation of 10°, α = 0.05 and β = 0.10, a minimum of 17 individuals was determined for each group.

The mean of three trials was used for the statistical analysis of the kinematic, kinetic and EMG data. Sample characteristics, kinematic, kinetic and IEMG data were screened for normality assumptions using the Shapiro Wilk test. Independent t-tests were used to compare sample characteristics. The kinematic, kinetic and EMG variables were compared between groups using three separate sets of multivariate analysis of variance tests. In the occurrence of multivariate effects, univariate effects were tested for all variables of interest. The significance level was set to 5% (*P*<0.05). Cohen's d effect size measurements were calculated and defined as follows <0.2 =  trivial, 0.2 to 0.5 =  small, 0.5 to 0.8 =  medium and >0.8 =  large [42]. All statistical comparisons were performed using SPSS, version 15.0 (SPSS Inc., Chicago, IL).

## Results

The multivariate analysis of variance tests revealed significant differences between the groups regarding the kinematic [F(10,29) = 24.8, *P*<0.001; Wilk's λ = 0.105] and kinetic [F(6,33) = 35.0, *P*<0.001, Wilk's λ = 0.136] and EMG [F(4,35) = 10.48, *P*<0.001, Wilk's λ = 0.455] data. The results of the univariate analysis of variance are summarized below and can be seen in Table 2.

**Table 2:** Comparison of kinematic data, peak joint angles (°), kinetic data, internal joint moments (Nm/Kg) and EMG data, eccentric phase muscle activation (% MVIC) between patellofemoral pain group and control group during propulsion phase for SLTHT.

	Control Group	Patellofemoral Group	P-value	Effect size
**KINEMATIC DATA**				
Trunk lean*	6.5 (4.4)	(-)5.6 (4.7)	<0.001	2.6
Trunk flexion	8.8 (3.4)	14.3 (4.7)	<0.001	1.3
Contralateral pelvic drop	7.4 (2.3)	14.8 (3.1)	<0.001	2.7
Hip internal rotation	10.4 (2.0)	18.9 (5.7)	<0.001	1.9
Hip adduction	13.0 (3.9)	19.3 (3.7)	<0.001	1.7
Hip flexion	71.1 (7.3)	80.9 (4.8)	<0.001	1.6
Knee abduction	6.6 (3.6)	7.3 (3.2)	0.518	0.2
Knee flexion	66.0 (3.5)	63.8 (3.4)	0.053	0.6
Ankle pronation	40.4 (4.4)	50.9 (7.4)	<0.001	1.7
Ankle dorsiflexion	40.1 (6.2)	35.6 (4.5)	0.011	0.8
**KINETIC DATA**				
Hip abductor	1.1 (0.3)	1.7 (0.4)	<0.001	1.7
Hip extensor	3.8 (1.1)	1.8 (0.8)	<0.001	2.1
Knee abductor	1.1 (0.2)	2.6 (0.5)	<0.001	3.9
Knee extensor	1.2 (0.4)	0.9 (0.2)	0.006	0.9
Ankle supinator	0.4 (0.2)	0.7 (0.3)	0.002	1.2
Ankle plantar flexor	2.4 (0.5)	2.0 (0.6)	0.018	0.7
**EMG DATA**				
Gluteus maximus	10.1 (9.3)	20.4 (11.9)	0.005	1.0
Gluteus medius	10.5 (10.1)	20.9 (10.0)	0.002	1.0
Biceps femoris	8.5 (12.5)	15.9 (7.2)	0.026	0.7
Vatus lateralis	8.6 (6.2)	37.4 (19.3)	<0.001	2.0

Data expressed as mean (standard deviation).

*Negative value: ipsilateral trunk lean. Abbreviations: SLTHT =  single-leg triple-hop test.

### Kinematic and kinetic data

#### Trunk

A significant difference was found between the groups for trunk lean. PFG and CG exhibited ipsilateral and contralateral trunk lean, respectively (mean difference: 12.0°; 95% confidence interval [CI]: 9.1 to 14.9°). Flexion was greater in the PFG (mean difference: 5.4°; 95% CI: 2.8 to 8.1°).

#### Pelvis

A greater contralateral pelvic drop was found in the PFG (mean difference: 7.5°; 95% CI: 5.7 to 9.2°).

#### Hip

The PFG exhibited greater internal rotation (mean difference: 8.6°; 95% CI: 5.9 to 11.3°), flexion (mean difference: 9.8°; 95% CI: 5.9 to 13.8°) and adduction (mean difference: 6.2°; 95% CI: 3.8 to 8.7°). The internal abductor moment was greater (mean difference: 0.6 Nm/Kg; 95% CI: 0.3 to 0.8 Nm/Kg) and the internal extensor moment was lower (mean difference: 2.0 Nm/Kg; 95% CI: 1.4 to 2.6 Nm/Kg) in the PFG.

#### Knee

No statistically significant differences were found between the groups regarding abduction (mean difference: 0.7°; 95% CI: (-) 1.5 to 2.9°) or flexion (mean difference: 2.2°; 95% CI: 0.0 to 4.4°). The PFG exhibited greater internal abductor moment (mean difference: 1.5 Nm/Kg; 95% CI: 1.3 to 1.8 Nm/Kg) and lower internal extensor moment (mean difference: 0.3 Nm/Kg; 95% CI: 0.1 to 0.5 Nm/Kg).

#### Ankle

A significant difference was found between the groups. Pronation was greater (mean difference: 10.4°; 95% CI: 6.5 to 14.3°) and dorsiflexion was lower (mean difference: 4.5°; 95% CI: 1.1 to 8.0°) in the PFG. A greater internal supinator moment (mean difference: 0.3 Nm/Kg; 95% CI: 0.1 to 0.4 Nm/Kg) and a lower internal plantar flexor moment (mean difference: 0.4 Nm/Kg; 95% CI: 0.1 to 0.7 Nm/Kg) were found in the PFG.

### EMG data

A significant difference was found between the groups. More EMG activation was observed for all of the muscles analyzed when the PFG was compared with the CG during the propulsion phase of the SLTHT, with more activation found in the PFG: Gluteus Maximus (GM) had 10.2% MVIC.s (95% CI: 3.4 to 17.1); Gluteus Medius (GMed) had 10.4% MVIC.s (95% CI: 4.0 to 16.9); Biceps Femoris (BF) had 7.4% MVIC.s (95% CI: 0.9 to 14.0); and Vastus Lateralis (VL) had 28.9% MVIC.s (95% CI: 19.7 to 38.0) more activation was found in the PFG.

## Discussion

This study investigated biomechanical differences in the propulsion phase of the SLTHT between women with and without PFPS. The findings confirm the hypothesis that women with PFPS exhibit distinct biomechanical alignment of the trunk and lower limb during the propulsion phase of this test, which is in agreement with data described in previous studies involving squatting tasks [10], [11].

In the present study, the PFG exhibited greater angular values of ipsilateral trunk lean (216%), trunk flexion (38%), contralateral pelvic drop (50%), hip internal rotation (45%), hip adduction (32%), hip flexion (12%), ankle pronation (21%) and lower ankle dorsiflexion (11%) than the pain free controls. The ipsilateral trunk lean may be an attempt to minimize the excessive pelvic drop and the consequent excessive hip adduction [43], [44]. Nakagawa et al. [18] found an increase in ipsilateral trunk lean and pelvic drop in women with PFPS with the knee flexed at 60° during lower limb weight-bearing activities. In addition, the greater hip internal rotation and adduction may contribute to the increase in PF pain, since these factors affect the PF joint contact area and PF stress [8], [9], [45]. Moreover, a reduction in ankle dorsiflexion may contribute to the altered kinematics of the lower limb, increasing dynamic knee valgus and ankle pronation [46].

Reduced PF compression may potentially occur when the mechanical demand on the quadriceps is lower. During weight-bearing activities of the lower limbs, the hip extensors and ankle plantar flexors act eccentrically in synergy with the knee extensors. Thus, less mechanical demand occurs on the quadriceps when there is a greater contribution from these muscles [43], [47]. The position of the trunk is also an important strategy to minimize the demands imposed on the quadriceps, since a flexed trunk increases the demand on the hip extensors and decreases the demand on the knee extensor [30], [43].

The internal joint moments that oppose external joint moments generated by the execution of the SLTHT, contribute to a deficient dynamic alignment of the lower limb and, possibly, increase PF stress. This confirms the hypothesis that women with PFPS have greater internal hip abductor (35%) and ankle supinator (42%) moments and a lower internal knee extensor moment (25%). However, the greater internal knee abductor (58%) moments and lower internal hip extensor (52%) and ankle plantar flexor (17%) moments do not confirm the initial hypothesis.

The lower internal hip, knee and ankle extensor moment in conjunction with excessive trunk flexion may be an attempt to diminish the recruitment of the quadriceps and minimize PF stress. The greater VL EMG activity could be a compensatory strategy that may increases PF stress [48]. In addition, the greater BF activation in the PFG may also be related to a possible increase in PF stress [20]. Therefore, since the EMG analysis demonstrated greater activity in the PFG in all of the muscles assessed (50% for the GM and GMed, 47% for the BF and 77% for the VL). The hypothesis of lower VL and BF activation was not confirmed.

The present findings are in agreement with data described by Souza and Powers [44], who found increased GM activity during the step-down test and related this finding to a possible attempt to contain the deficient lower limb alignment. The GM is an important hip extensor and lateral rotator. Therefore, more EMG activity in the GM would avoid the greater hip internal rotation and assist the quadriceps in the SLTHT execution.

The PFG exhibited greater internal hip abductor and ankle supinator moments, which may contribute to avoiding excessive pelvic drop/hip adduction and ankle pronation, respectively. Even with ipsilateral trunk lean, the center of mass was not sufficiently projected laterally to the knee to control biomechanical alignment. Thus, the greater GMed activity in the PFG could be to stabilize the lower limb. Bolgla et al. [49] described a similar finding during the step-down test. However, Nakagawa et al. [17] and Aminaka et al. [50] found lower activation of the GMed during a single-leg squat. Therefore, further investigations are needed.

The recruitment of the muscles analyzed herein seems to be a compensatory strategy for the deficient dynamic alignment in the high-demand tasks placed on the knee during the SLTHT [26]. The propulsion phase should be considered in studies involving jumping activities and the assessment of individuals with PFPS, as this task exhibits impairments in biomechanical mechanisms that may influence the pathogenesis of PFPS and could be affected by anterior knee pain.

Regarding the most important findings of the present study, restoring lower limb dynamic alignment during the propulsion phase and minimizing the mechanical demands on the quadriceps are clinically relevant and should be directed mainly at the trunk and hips, possibly through muscle strengthening activities.

Numerous studies have assessed the jump upon landing by considering this task the most dynamic and greatest impact on the joint. However, the present study showed that the mechanisms that make up the malalignment of the lower limb are also present in the propulsion phase. As yet, it is impossible to say which of the mechanisms, propulsion or landing, are more important to an understanding of the syndrome.

Based on a patellofemoral pain consensus statement from the 3^rd^ International Patellofemoral Pain Research Retreat [51] more comprehensive approach to muscle activation and studies to compare mechanics across a variety of tasks, to help define the tasks likely to reveal abnormal mechanics should be considered in the design of future studies.

Studies of minimal clinically important differences regarding kinematic variables of the lower limbs should be carried out to allow better estimates of the results and clinical interpretations of a given form of treatment. As cause and effect cannot be established in a cross-sectional design, this relationship should be investigated in future studies.

The present study has limitations that should be considered. PF pain and muscle strength were not assessed during the SLTHT, which could have influenced the biomechanical pattern of the lower limb [52] and could be correlated with the results. Ankle pronation is a variable that should be investigated further with a more robust foot model, rather than the simple biomechanical foot model used in the present study. The velocity of the test was not controlled in order to avoid influencing the performance of each individual. However, this may have affected the quality of the EMG signal. Moreover, the velocity at which the transition between knee flexion and extension occurs may facilitate the contraction of the quadriceps [47]. Upper limb swing may help to increase the jump performance [38], [39], [53]. Our intention was to avoid arm movements influencing the jump data.

## Conclusions

Ipsilateral trunk lean, excessive contralateral pelvic drop, hip adduction and internal rotation are associated with abnormal movement patterns in women with PFPS. Increased trunk flexion, internal hip abductor moment, internal ankle supinator moment and greater GM and GMed EMG activity seem to be control strategies for a deficient dynamic alignment of the lower limb and trunk. In addition, the lower contribution of the internal hip extensor and ankle plantarflexor moments to the internal knee extensor moment, together with greater VL and BF EMG activity, could increase PF stress in women with PFPS due to an excessive load on the quadriceps.
